# Effects of Changes in Multiple Chronic Conditions on Medical Costs among Older Adults in South Korea

**DOI:** 10.3390/healthcare10040742

**Published:** 2022-04-15

**Authors:** Soojin Park, Jin Young Nam

**Affiliations:** Department of Healthcare Management, Eulji University, Sungnam-si 13135, Korea; sj_eu@naver.com

**Keywords:** multiple chronic conditions, out-of-pocket costs, medical expenses, cognitive decline, aging society

## Abstract

This study aims to analyze the relationship between cognitive function and out-of-pocket cost of the state change of multiple chronic conditions in individuals aged 60 or older. Data from the 2014 to 2018 Korean Longitudinal Study of Aging were used for 2202 older adults who were cognitively “normal” at the start of the survey. Four status change groups were established (“Good → Good,” “Good → Bad,” “Bad → Good,” and “Bad → Bad”) according to the change in the number of chronic diseases. Generalized estimating equation modeling analyzed the association between these changes and out-of-pocket medical cost. Out-of-pocket cost was significantly higher among older adults with multiple chronic conditions (*p* < 0.0001). Total out-of-pocket medical cost and out-of-pocket cost for outpatient care and prescription drugs were significantly higher for Bad→ Bad or Good → Bad changes. Older adults with cognitive decline had significantly higher total out-of-pocket medical cost and out-of-pocket cost for prescription drugs. This study demonstrates the need to improve the multiple chronic conditions management construction model to enhance the health of older adults in Korea and secure national health care finances long-term. It provides a foundation for related medical and medical expenses-related systems.

## 1. Introduction

A common public health issue around the world is aging. In south-east Asia, it has been reported that the pace of acceleration of aging along with the decline in fertility rates is faster than in other countries [1]. As the world becomes an aging society, various public health problems arise with increased geriatric diseases and, thus, medical expenses for older adults [2]. Twenty-three percent of the global disease burden is attributable to disability in those aged 60 and over, and chronic non-communicable diseases (NCDs) account for a high proportion [3]. Recently, the World Health Organization (WHO) also introduced the concept of “best buy” to expand its core intervention package for NCDs for targeting low/middle income countries (LMICs) [4]. According to previous research, population aging has significantly increased disease burden among older adults for age-dependent disabilities (cardiovascular, chronic obstructive pulmonary disease, diabetes, and neurological disorders) [2,3].

As the number of chronic diseases increases, the proportion of people with multimorbidity, in which one person has two or more chronic diseases, has also increased [5]. Older adults in South Korea have an average of 4.1 chronic diseases [6], and the number of multiple chronic conditions (MCCs) gradually increased from 14.9% in 2012 to 17.8% in 2018 [7]. People with two or more chronic diseases face increased medical costs and health care utilization [5], poor quality of life [8], deterioration of physical function, and disability compared to those with only one [9]. According to previous studies, people with one or no chronic disease receive an average of 11.7 treatments a year while those with MCCs receive an average of 18.3, which is socially burdensome for medical expenses [10]. Bähler et al. (2015) in Switzerland estimated that the average total medical cost of elderly patients with MCCs was 5.5 times higher than for those without [11].

Along with chronic diseases, many representative geriatric diseases involve cognitive decline, such as mild cognitive impairment or dementia. Dementia affected approximately 50 million people worldwide in 2018, which is projected to increase to approximately 150 million by 2050 [2]. Lee and Cho found that people with MCCs have a higher risk of cognitive decline than those with only one [12]. Older adults with cognitive impairment spent more on inpatient treatment due to long-term hospitalization than on outpatient treatment [13], and, at the stage of cognitive decline, the expenditure on prescription drugs due to drug use was higher [14].

This study aimed to analyze the association of changes in MCC status with out-of-pocket medical cost using longitudinal data on Korean elderly people over four years (2014–2018) with a subgroup analysis of whether cognitive function declines. Our findings emphasize the need for effective management of MCCs to identify factors affecting older adults’ out-of-pocket expenses and reduce the burden.

## 2. Materials and Methods

### 2.1. Study Participants and Database Information

**Figure 1:**
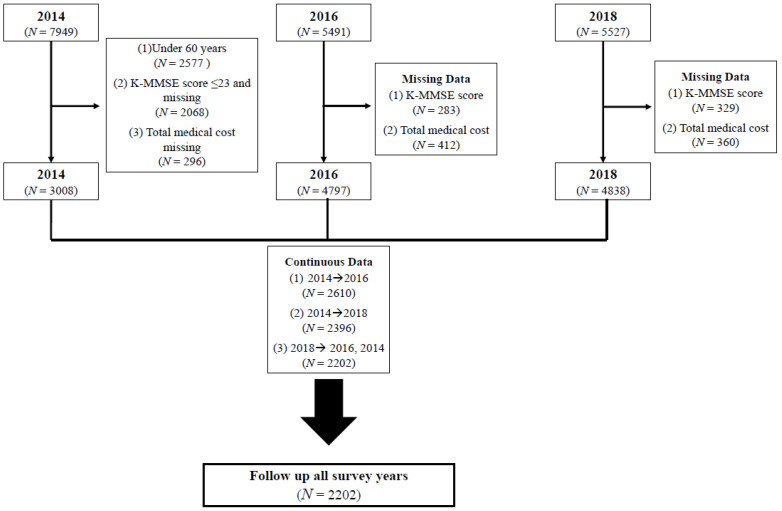
Flow chart representing the selected study population.

This study used raw data from a longitudinal study—the Korea Longitudinal Study of Aging (KLoSA)—in 2014, 2016, and 2018 (Figure 1). The KLoSA was conducted by the Ministry of Labor of Korea and is a nationally representative panel survey using a computer-assisted personal interviewing system. The survey participants were community-dwelling South Korean adults aged 45 years and older. Participants were randomly selected using a multistage stratified sampling method, sorting by residential type in the order of administrative codes. Skilled interviewers used a structured questionnaire seeking data pertaining to demographics, family, health, employment, healthcare utilization, and so on. We used the original panel data in 2014, 2016, and 2018 for analysis. More details about the KLoSA can be found at https://survey.keis.or.kr/eng/index.jsp (accessed on 14 March 2021).

First, we excluded participants who (a) were aged <60 years, (b) had low cognitive impairment (Korean Mini-Mental State Examination score <24), and (c) were missing data on medical cost in 2014, 2016, and 2018. Additionally, only those who participated in the 2014, 2016, and 2018 surveys were included. For the final analysis, we had 2202 participants.

### 2.2. Medical Cost

Medical expenses were calculated by summing admission medical, outpatient medical, visit treatment, and regular prescription drug expenses. The medical expenses investigated by the KLoSA are out-of-pocket expenses with responses based on the respondent’s recollection.

### 2.3. Changes in MCCs

The main predictor variable was change in individual status of having MCCs between any two waves of the KLoSA. The chronic diseases assessed in the KLoSA were hypertension, diabetes, cancer, chronic obstructive pulmonary disease, liver disease, heart disease, cerebrovascular disease, psychiatric disease, and arthritis. The presence of each chronic disease was assessed by asking the participant whether they had been diagnosed by a physician or were currently under treatment (e.g., taking medication) for these diseases. Participants with more and less than two chronic diseases were defined as having a “bad (more than two chronic diseases)” or “good (0–1 chronic disease)” medical condition, respectively. In this study, MCCs were defined in four categories: “Good → Good”; “Good → Bad”; “Bad → Good”; or “Bad → Bad.”

### 2.4. Covariates

The covariates assessed in this study were gender (men or women), age (60–69, 70–79, ≥80 years), marital status (married or unmarried, including divorced, widowed, and separated), education level (less than middle school/more than high school), employment status (employed or unemployed), household income (discretized based on quartiles), and region (city/rural). To quantify health-related covariates, body mass index (BMI) was classified as underweight (0–18.4 kg/m^2^), normal (18.5–24.9 kg/m^2^), or obese (≥25 kg/m^2^). We also included individuals’ current smoking status (yes or no), alcohol consumption (yes or no; defined either as three drinks per day or seven drinks or more per week for alcohol such as soju, beer, wine, whiskey, and raw rice wine), and self-rated health (good: “very good,” “good,” or “fair”; poor: “poor” or “very poor”), regular physical activity (yes or no; defined as any type of exercise at least once a week), activities of daily living (ADL)/instrumental ADL (IADL) status (none: score of 0 on both ADL and IADL; mild: score of 0 on ADL and 1 or more on IADL; and severe: score of 1 or more on both ADL and IADL).

### 2.5. Statistical Analysis

We aimed to analyze if the distributions for out-of-pocket medical expenses were highly skewed, so we used the Kruskal–Wallis test, a non-parametric test of rank. We used a generalized estimating equation (GEE) model with odds ratios (ORs) and 95% confidence intervals (CIs) with log link, γ distribution, and robust standard errors to examine the relationship between MCCs and medical cost, adjusted for all covariate variables. A GEE model with log link was also performed for subgroup analysis by cognitive status. All analyses were performed using SAS software, version 9.4 (SAS, Inc., Cary, NC, USA). All *p*-values < 0.05 were considered statistically significant.

## 3. Results

Table 1 presents the average out-of-pocket medical costs at baseline in 2014 using Kruskal–Wallis analysis. The average out-of-pocket total medical costs were 1163.8 USD for participants with MCCs and 456.1 USD for those without MCCs, which was a statistically significant difference (*p* < 0.00001). There were significant differences in average cost for out-of-pocket total medical costs according to age, educational level, household income, BMI, self-rated health, current smoking, current alcohol consumption, and ADL/IADL status (*p* < 0.05; Table 1).

Table 2 shows the association between change in MCCs and out-of-pocket total, hospital admission, outpatient, and pharmacy medical cost after adjusting for socioeconomic status using GEE analysis. Compared to the reference group (Good → Good), the change in MCCs significantly increased the total, outpatient, and pharmacy out-of-pocket medical costs in Bad → Bad and Good → Bad groups (*p* < 0.0001). However, there was no significant association between change in MCCs and out-of-pocket hospital admission medical costs. In terms of cognitive function, the out-of-pocket total and pharmacy medical cost were significantly higher for those with cognitive impairment than those with normal cognitive function (Total: β = 0.2156, *p* = 0.0079; pharmacy: β = 0.2143, *p* < 0.0001). In terms of out-of-pocket total and pharmacy medical costs, women’s medical costs were statistically significantly lower than men’s (Total: β = −1.1786, *p* = 0.0051; pharmacy: β = −0.2498, *p* < 0.0001; Table 2).

## 4. Discussion

This study analyzed the associations of out-of-pocket costs for total medical cost, inpatient care, outpatient care, and prescription drugs with changes in MCC status. From the perspective of purchasing power parity (PPP), not only Korea but also BRICs countries such as Brazil, Russia, India, South Africa, and China reported that medical expenses per capita will increase [15]. In the long run, medical expenses for MCCs are needed because out-of-pocket medical expenses due to chronic diseases can plunge about 150 million people worldwide into poverty [16].

Changes in MCC status influenced all out-of-pocket medical costs (total, outpatient care, prescription drugs) with the exception of inpatient care. Total out-of-pocket medical cost and out-of-pocket cost for outpatient care and prescription drugs significantly increased among those whose MCCs changed from Bad to Bad or Good to Bad compared to the reference group (Good → Good). Additionally, we adjusted for cognitive function status and observed that total out-of-pocket cost and out-of-pocket cost for prescription drugs were significantly higher among older adults with cognitive decline than those with normal cognitive function.

In this study, total out-of-pocket medical cost was lower among women than men. Previous studies reported that MCCs common among men are chronic obstructive pulmonary disease, cardiovascular disease, hypertension, and heart failure while those common among women are mental disorders (e.g., depression, anxiety), arthritis, and varicose veins; in other words, the MCCs that are more prevalent among men are associated with greater medical needs and prescription cost [17,18,19]. Our result that total out-of-pocket medical cost and out-of-pocket cost for prescription drugs are significantly lower among women than men is consistent with previous reports.

Prescription drugs used to treat cognitive decline are generally costly, and polypharmacy is often necessary for older adults because they require concurrent treatments for a number of conditions, including fall, depression, and MCCs [20]. In addition, previous findings that individuals with MCCs require the use of polypharmacy for their conditions and that such use contributes to cognitive decline as an adverse drug reaction highlights a significant correlation between MCCs and cognitive decline [21]. Furthermore, the severity of cognitive impairment is reportedly affected by the cost per drug and number of drugs used [14], calling for further research on the prescription drug costs according to the severity of cognitive impairment.

On the other hand, we observed no significant association between changes in MCC status and out-of-pocket cost for inpatient care. Contradicting our findings, Leibson et al. (2015) reported that the cost of inpatient care increases while the cost of outpatient care decreases among those with cognitive impairment [13]. This discrepancy may be due to the differences in the medical cost reimbursement system and healthcare systems across countries and the changes in the patterns of healthcare utilization among older adults. One Korean study reported that utilization of long-term care hospitals increased among the middle elderly [22]. Further, the cost of long-term hospital care was the highest among those with physical and cognitive functional impairment [22]. The rate of long-term care hospital admission is anticipated to continue to rise in the future owing to a variety of factors, including increased needs due to the growing elderly patient population and the delivery system provided by long-term care hospitals [22]. However, one limitation of this study is that we could not analyze the cost of long-term hospital care. Thus, subsequent studies should include long-term hospital cost in the analysis of medical cost among older adults.

There was no interaction term, only MCC status changes, and the medical cost for all variables increased when the status changed from Bad to Bad or from Good to Bad, suggesting that older adults and their families must pay more attention to the treatment of MCCs and that healthcare policies must be updated (Supplementary Table S1). As our results show, MCCs have a substantial impact on increased medical cost and thus present complicated medical needs, and an effective healthcare delivery system must be established to properly address these conditions. In addition, multidisciplinary approaches or self-care education for lifestyle modification should be implemented to promote appropriate management of conditions among patients diagnosed with multiple diseases and treatment needs [23]. Education and management of polypharmacy is also essential for patients with MCCs, and, based on a previous report that medication self-efficacy and education about medication administration substantially contribute to medication adherence, systematic education about medication administration should be implemented [24].

While it has been reported that medical expenditure increases with age [25], our results showed that total out-of-pocket medical cost decreased with advancing age. Hazra et al. (2018) similarly reported that total out-of-pocket medical cost significantly decreased with advancing age. Consistent with our findings, annual medical expenditure decreased with increasing age, and the decline was more dramatic when including MCCs and disabilities [26].

The reason for the inconsistency in study findings, including ours, pertains to the limitation in comparing results due to the lack of a clear definition and criteria for MCCs as well as the disparity in healthcare delivery systems and medical fee reimbursement systems across countries [27]. Having patients with MCCs be treated separately by different specialists for each of the conditions undermines treatment continuity, which, in turn, elevates the risk of overtreatment, undertreatment, and mistreatment. Hence, a new healthcare system and management that can promote continuity of care are needed [25,28].

Total out-of-pocket medical cost was also higher among unemployed and obese individuals, those with a poor self-rated health, and those with poor ADL/IADL, and lower among those with low income. Fostering a supportive environment that promotes health behaviors among older adults to lower the risk of a single chronic condition as well as MCCs is recommended [7,29]. Lifestyle modification such as avoiding heavy drinking and smoking and practicing a healthy diet and physical activities can help lower risks and prevent some of the highly prevalent diseases, namely hypertension, diabetes, and cardiovascular diseases.

This study has a few limitations. Older adults with MCCs have been reported to frequently utilize the emergency department (ED) and, thus, have high medical expenditure [30]. The KLoSA does not investigate medical cost for ED care, so we could not examine these data in this study. Further, while many studies have analyzed the direct cost of MCCs, study data on indirect cost of MCCs are relatively lacking. We were also unable to estimate indirect cost, such as productivity loss. Subsequently, studies should address these limitations and investigate various types of medical costs. Despite these limitations, however, this study sheds light on dynamic associations among the study variables by using longitudinal as opposed to cross-sectional data, which only allows an examination of a static relationship and hinders establishing causality. However, due to the nature of secondary data analysis, there may be uncontrolled confounders caused by limitations with the survey. Another drawback of panel data is that missing data may be present during repeated measurements of individuals. However, to enhance the accuracy of our analysis, we only included participants who completed all rounds of the survey. Additionally, the significance of this study lies in our examination of individuals aged 60 years and over—a broader age group and one potentially at risk for cognitive impairment compared to the ≥65 group that has been predominantly studied in existing literature on cognitive impairment and total out-of-pocket medical cost. This also suggests that our findings can serve as additional evidence on whether MCCs affect medical cost.

Even in countries with very similar population distribution and health systems, long-term medical expenditures have various outcomes. This leads to lower credibility of comparability of cross-border health expenditure studies even within Organization for Economic Cooperation and Development (OECD) countries [15,16]. Among the BRICs countries, China is also expected to significantly expand investment in the health sector among Association of Southeast Asian Nations (ASEAN) regions in the future [31]. Therefore, Korean policy makers need to find a direction by referring to health programs for the elderly in various countries as well as in South Korea. [15].

Systems or programs that help older adults practice health behaviors are needed, and, for older elderly individuals who cannot engage in such practices independently, measures must be devised to promote their families and supporting organizations to reduce risk factors and practice health behaviors. According to previous studies, technologies and platforms that access drug use management through smartphones or tablets have recently been implemented [32]. Through online intervention, users were able to manage drugs at an appropriate level, and awareness of drug use increased [32]. If the latest technology is stabilized in the future, it might be possible to efficiently manage drug use through the families of elderly patients with MCCs. The current system for chronic disease management lowers the out-of-pocket cost for registered patients and provides incentives based on quality and a chronic disease management fee for healthcare providers [23]. However, healthcare facilities that use a fee-for-service system are likely to not prefer providing care for patients with MCCs, so there is a pressing need for discussions about adjusting the fees for patients with complicated and severe conditions, such as MCCs.

## 5. Conclusions

This study aims to investigate the effects of changes in MCC status on out-of-pocket medical costs. There was no significant association between changes of MCC status and out-of-pocket cost for inpatient care, but the out-of-pocket cost for outpatient care and prescription drugs were significantly higher among patients whose MCC statuses were consistently bad and or whose statuses changed from good to bad.

Patients with MCCs with high out-of-pocket medical cost are bound to experience a deterioration in quality of life and self-rated health [8], and these costs can also contribute to physical and mental impairment, highlighting the importance of management [33]. In order to reduce the prevalence of NCDs, strategic health promotion should be valued and the importance of primary care that can provide for early detection and management should be emphasized [16]. Additionally, in terms of medical costs, it is necessary to find a way to encourage primary care and implement the audit system together while providing incentives along with the reorganization of the medical expenses payment system [16].

Employing single-disease-targeting approaches to manage older adults with MCCs is ineffective, and a more systematic and comprehensive approach is required [28]. In particular, older adults with cognitive decline and poor MCC status have high out-of-pocket cost for prescription drugs because polypharmacy is essential to treat MCCs. Thus, periodic, systematic management is essential to minimize any adverse reactions due to polypharmacy [24].

Furthermore, based on these findings, more specific studies on productivity losses caused by MCCs should be conducted by examining not only direct medical costs but also indirect medical costs.

## Figures and Tables

**Table 1:** Differences in total medical cost according to the characteristics of the study population at baseline (Unit: USD ^1^).

	2014		
	Mean	SD ^2^	*p*-Value
Multiple chronic conditions			
Yes	1163.8	1839.2	<0.0001
No	456.1	942.6	
Cognitive impairment			
No (Normal)	621.6	1257.6	
Yes	-	-	
Sex			
Men	659.1	1451.7	0.2163
Women	582.6	1016.5	
Age			
60–69	566.6	1047.6	<0.0001
70–79	685.7	1485.7	
80+	707.3	1418.7	
Educational level			
Less than middle school	616.0	1091.6	0.0023
High school or more	630.7	1485.5	
Region			
City	645.1	1318.2	0.3740
Rural	551.8	1055.3	
Employment status			
Employed	592.9	1208.6	0.4199
Unemployed	655.8	1316.5	
Household income			
1Q	791.0	1439.1	0.0002
2Q	562.4	944.4	
3Q	580.0	1236.6	
4Q	602.9	1342.9	
Marital status			
Unmarried	597.3	1046.1	0.3130
Married	627.0	1299.7	
BMI			
Underweight	528.4	1127.1	<0.0001
Normal	569.5	1291.7	
Obesity	774.7	1165.5	
Self-rated health			
Good	325.6	750.5	<0.0001
Bad	798.2	1451.3	
Current smoking			
Yes	494.5	913.0	0.0013
No	641.8	1302.8	
Current alcohol consumption			
Yes	565.9	1100.6	0.0041
No	653.8	1339.2	
Physical activity			
Yes	637.5	1390.9	0.3376
No	610.8	1157.6	
ADL/IADL status			
None	605.1	1240.3	0.0008
Mild	774.4	1290.3	
Severe	1324.1	1552.1	
Mean (SD)	621.8	(1257.6)	
Median	252.1		
Total	136,875.6		

^1^ USD, US dollars in 2014; ^2^ SD, Standard deviation.

**Table 2:** The association between the change of multiple chronic diseases and total medical cost.

Out-of-Pocket Medical Cost								
	Total		Hospital admission		Outpatient		Pharmacy	
	β	*p*-value	β	*p*-value	β	*p-*value	β	*p*-value
Intercept	6.0634	<0.0001	7.7544	<0.0001	5.0688	<0.0001	5.9254	<0.0001
Change of multiple chronic diseases								
Bad → Bad	0.8048	<0.0001	0.1129	0.3925	0.5073	<0.0001	0.5907	<0.0001
Bad → Good	0.1133	0.1069	−0.1373	0.2682	0.1779	0.0854	0.0081	0.8884
Good → Bad	0.6737	<0.0001	0.1052	0.3829	0.4392	<0.0001	0.3938	<0.0001
Good → Good	ref.		ref.		ref.		ref.	
Cognitive impairment								
Yes	0.2156	0.0079	0.2002	0.2429	0.1284	0.0642	0.2143	<0.0001
No (Normal)	ref.		ref.		ref.		ref.	
Sex								
Men	ref.		ref.		ref.		ref.	
Women	−0.1786	0.0051	−0.0693	0.4804	−0.0108	0.8885	−0.2498	<0.0001
Age								
60–69	ref.		ref.		ref.		ref.	
70–79	−0.0834	0.1702	−0.0661	0.5565	0.0900	0.2187	−0.0530	0.1808
80+	−0.1480	0.1582	−0.1523	0.5243	0.0544	0.5833	0.0060	0.9374
Educational level								
Less than middle school	ref.		ref.		ref.		ref.	
High school or more	−0.0285	0.6397	−0.0187	0.8562	−0.1261	0.0509	−0.0295	0.5008
Region								
City	ref.		ref.		ref.		ref.	
Rural	0.0129	0.8301	0.2086	0.0276	0.0178	0.8078	−0.0012	0.9813
Employment status								
Yes	ref.		ref.		ref.		ref.	
No	0.1203	0.0214	0.0048	0.8946	0.0462	0.5179	0.0048	0.8946
Household income								
1Q (Lowest)	0.0141	0.8677	−0.2090	0.2002	−0.2214	0.0147	0.0670	0.2749
2Q	−0.1978	0.0040	−0.3547	0.0106	−0.2205	0.0032	0.0037	0.9442
3Q	0.0323	0.6546	−0.0004	0.9976	−0.0680	0.3898	0.0230	0.6159
4Q (Highest)	ref.		ref.		ref.		ref.	
Marital status								
Unmarried	−0.0059	0.9384	0.1015	0.4992	−0.0633	0.4652	−0.0927	0.0843
Married	ref.		ref.		ref.		ref.	
BMI								
Underweight	0.0735	0.5818	−0.0877	0.6712	−0.0558	0.6811	0.0340	0.7335
Normal	ref.		ref.		ref.		ref.	
Obesity	0.1423	0.0118	−0.0900	0.3548	0.1173	0.0912	0.0687	0.0756
Current smoking								
Yes	−0.1931	0.0252	−0.0305	0.8350	−0.1420	0.0317	−0.1022	0.1267
No	ref.		ref.		ref.		ref.	
Current alcohol consumption								
Yes	−0.0960	0.0731	−0.2153	0.0168	−0.1586	0.0053	−0.0273	0.5642
No	ref.		ref.		ref.		ref.	
Self-rated health								
Good	ref.		ref.		ref.		ref.	
Bad	0.6927	<0.0001	0.2899	0.0126	0.4164	<0.0001	0.2923	<0.0001
Physical activity								
Yes	ref.		ref.		ref.		ref.	
No	−0.0769	0.0295	0.1476	0.1011	−0.0617	0.2462	−0.0769	0.0295
ADL/IADL status								
None	ref.		ref.		ref.		ref.	
Mild	0.2908	0.0674	0.4331	0.0482	0.1846	0.2518	−0.1057	0.0905
Severe	1.0950	<0.0001	0.6426	0.0018	0.6496	0.0045	−0.3490	0.0013

## Data Availability

The data that support the findings of this study are openly available in “KLoSA” at https://survey.keis.or.kr/eng/index.jsp (accessed on 14 March 2021).

## Supplementary Materials

The following supporting information can be downloaded at: https://www.mdpi.com/article/10.3390/healthcare10040742/s1, Supplementary Table S1: Subgroup analysis for the association between the change of multiple chronic conditions and total medical cost.
