# Supplementary material for: Effects of Changes in Multiple Chronic Conditions on Medical Costs among Older Adults in South Korea
Source: Healthcare (Basel). 2022 Apr 15;10(4):742. doi: 10.3390/healthcare10040742 (PMC9029782; doi:10.3390/healthcare10040742)
Supplement: Supplementary file 1 [file healthcare-10-00742-s001.zip › healthcare-1629333-supplementary.pdf]

healthcare MDPI

Table S1. Subgroup analysis for the association between the change of multiple chronic conditions and total medical cost

	Change of multiple chronic conditions							
	Bad→Bad		Bad→Good		Good→Bad		Good→Good	P*
	β	P-value	β	P-value	β	P-value	β	
Sex								0.7972
Men	0.8810	<.0001	0.0606	0.5243	0.7242	<.0001	ref.	
Women	0.6936	<.0001	0.0136	0.9004	0.5093	<.0001	ref.	
Age								0.0883
60-69	0.7226	<.0001	-0.0785	0.4651	0.4764	<.0001	ref.	
70-79	0.8602	<.0001	0.0592	0.5626	0.7527	<.0001	ref.	
80+	0.6774	<.0001	0.3787	0.0177	0.7730	0.0047	ref.	
Education								0.5931
Less than middle school	0.7857	<.0001	-0.0279	0.7622	0.6205	<.0001	ref.	
High school or more	0.7715	<.0001	0.1147	0.3069	0.7334	<.0001	ref.	
Region								0.3784
Rural	0.6400	<.0001	-0.0218	0.8871	0.6975	<.0001	ref.	
City	0.8332	<.0001	0.0518	0.5175	0.6307	<.0001	ref.	
Employment status								0.7453
Unemployment	0.8153	<.0001	0.0410	0.5890	0.6330	<.0001	ref.	
Employment	0.7439	<.0001	0.0605	0.6322	0.7493	<.0001	ref.	
Household income								
1Q	0.9096	<.0001	-0.0743	0.5622	0.5221	0.0001	ref.	0.4928
2Q	0.9467	<.0001	0.1775	0.1971	0.6630	<.0001	ref.	
3Q	0.7634	<.0001	0.0970	0.5290	0.8791	<.0001	ref.	
4Q	0.6430	<.0001	-0.0546	0.6150	0.4917	0.0003	ref.	
Marital status								0.6316
Unmarried	0.8243	<.0001	0.0936	0.5200	0.7511	<.0001	ref.	
Married	0.7567	<.0001	0.0202	0.8117	0.5798	<.0001	ref.	
BMI								0.1567
Underweight	1.0224	0.0034	0.1703	0.5801	0.8384	0.0020	ref.	
Normal	0.8489	<.0001	0.0257	0.7711	0.7602	<.0001	ref.	
Obesity	0.6609	<.0001	0.0618	0.6256	0.4150	<.0001	ref.	

	Change of multiple chronic conditions							
	Bad→Bad		Bad→Good		Good→Bad		Good→Good	P^*
	β	P-value	β	P-value	β	P-value	β	
Self-rated health								0.0699
Good	0.8647	<.0001	0.0429	0.6556	0.9993	<.0001	ref.	
Bad	0.7388	<.0001	0.0547	0.5745	0.5572	<.0001	ref.	
Current smoking status								0.9451
Yes	0.6156	<.0001	0.1260	0.6227	0.6393	<.0001	ref.	
No	0.8040	<.0001	0.0284	0.7003	0.6539	<.0001	ref.	
Current alcohol consumption								0.3484
Yes	0.6764	<.0001	0.0481	0.6861	0.7175	<.0001	ref.	
No	0.8278	<.0001	0.0594	0.4943	0.6385	<.0001	ref.	
Regular physical activity								0.8995
Yes	0.8601	<.0001	0.0957	0.3491	0.7390	<.0001	ref.	
No	0.7449	<.0001	0.0064	0.9505	0.5853	<.0001	ref.	
ADL/IADL								0.9508
None	0.7966	<.0001	0.0275	0.7145	0.6143	<.0001	ref.	
Mild	0.8901	0.0026	0.2420	0.5353	0.8829	0.0039	ref.	
Severe	0.1456	0.7142	-0.0662	0.8954	0.0669	0.8513	ref.	

*P-interaction
